# Research Progress on Bioactive Factors against Skin Aging

**DOI:** 10.3390/ijms25073797

**Published:** 2024-03-28

**Authors:** Xin He, Xinyu Gao, Yifan Guo, Weidong Xie

**Affiliations:** 1 State Key Laboratory of Chemical Oncogenomics, Shenzhen International Graduate School, Tsinghua University, Shenzhen 518055, China; hexin22@mails.tsinghua.edu.cn (X.H.); gao-xy23@mails.tsinghua.edu.cn (X.G.); guo-yf23@mails.tsinghua.edu.cn (Y.G.); 2 Open FIESTA Center, Shenzhen International Graduate School, Tsinghua University, Shenzhen 518055, China; 3 Shenzhen Key Laboratory of Health Science and Technology, Institute of Biopharmaceutical and Health, Tsinghua University, Shenzhen 518055, China

**Keywords:** skin aging, biological activity factor, oxidative stress, collagen, polypeptide, enzyme

## Abstract

The relentless pursuit of effective strategies against skin aging has led to significant interest in the role of bioactive factors, particularly secondary metabolites from natural sources. The purpose of this study is to meticulously explore and summarize the recent advancements in understanding and utilization of bioactive factors against skin aging, with a focus on their sources, mechanisms of action, and therapeutic potential. Skin, the largest organ of the body, directly interacts with the external environment, making it susceptible to aging influenced by factors such as UV radiation, pollution, and oxidative stress. Among various interventions, bioactive factors, including peptides, amino acids, and secondary metabolites, have shown promising anti-aging effects by modulating the biological pathways associated with skin integrity and youthfulness. This article provides a comprehensive overview of these bioactive compounds, emphasizing collagen peptides, antioxidants, and herbal extracts, and discusses their effectiveness in promoting collagen synthesis, enhancing skin barrier function, and mitigating the visible signs of aging. By presenting a synthesis of the current research, this study aims to highlight the therapeutic potential of these bioactive factors in developing innovative anti-aging skin care solutions, thereby contributing to the broader field of dermatological research and offering new perspectives for future studies. Our findings underscore the importance of the continued exploration of bioactive compounds for their potential to revolutionize anti-aging skin care and improve skin health and aesthetics.

## 1. Introudution

Skin, as the body’s most direct organ in contact with the external environment [1,2], is not only a complex barrier organ [3] but also an intuitive manifestation of aging [4]. The degree of skin aging is not only a direct reflection of a person’s health status but has also been found to be an important indicator of predicting individual life expectancy [5]. Over time, skin can be affected by external and internal factors, such as light exposure, pollution, and disease, leading to aging. In the past, when discussing the causes and mechanisms of skin aging, it was found that oxidative stress theory, skin photoaging theory, inflammation theory, metabolism theory, etc., had a great influence on skin aging [6,7,8]. Because of the importance of oxidative stress in the skin aging process, most anti-skin-aging strategies and related products mainly focus on antioxidant defense. Previously, effective products were mainly derived from chemically extracted plant extracts and natural small molecule compounds.

In recent years, with the development of biotechnology and the research progress on skin structure and its function in medicine, people have gained a deeper understanding of the factors affecting skin aging and related mechanisms. In fact, the loss or destruction of skin structural proteins/lipids/sugars or production disorders caused by various factors will directly lead to skin aging. In addition, organelles in skin cells and biological macromolecules, such as proteins, lipids, polysaccharides, and nucleic acids, can interact with each other, and this process can accelerate skin aging. Hyaluronan (HA) is a nonsulfated glycosaminoglycan that has been widely used in biomedical applications. HA treatment of cells can enhance cell viability. Specifically in atrophic stressed cells, effective drugs containing HA revealed a noteworthy beneficial effect on the myogenic biomarkers indicating that it could be used as a promising platform for tissue regeneration, with specific attention to muscle cell protection against stressful agents [9]. Skin cells can communicate with related cytokines through exosomes. Meanwhile, skin cells can interact with external microorganisms, and these skin microorganisms, in turn, can interact with each other in a manner that directly or indirectly influences skin oxidative stress or inflammation of tissues involved in the skin aging process.

In conclusion, the primary aim of our study is to dissect the intricate role of bioactive factors, with a particular focus on secondary metabolites, in the fight against skin aging. As we delve into the realms of biotechnology and dermatological research, our understanding of the skin’s structural intricacies and functional dynamics continues to evolve, offering new horizons for anti-aging research. By compiling and summarizing recent advancements in the field, this study seeks not only to enrich the current academic discourse on skin aging and active ingredients but also to highlight potential pathways for the development of effective anti-aging interventions. Since many endogenous bioactive molecules, such as proteins, peptides, cytokines, and microbial metabolites, are themselves involved in the skin aging process, many bioactive components are directly used by pharmaceutical companies to develop relevant anti-skin-aging products. In addition, biological products also have the advantages of low environmental pollution, low side effects, high activity, specific targets, and easy biofabrication. Therefore, in recent years, the development of and research on biological anti-aging products in the skin, such as proteins, peptides, cytokines and microbial fermentation fluids, have developed rapidly as summarized below.

## 2. Protein-Derived Products

### 2.1. Collagen

Collagen is a fibrous protein found mainly in connective tissues, such as skin, bones, and joints. There are different types of collagen in the body, such as type I, type II, type III, type IV, and type V [10]. Type V collagen mainly acts on the tissues between the dermis and epidermis, and it can be used to maintain normal dermis–epidermal interactions [11]. It decreases over time, losing its original thickness and strength, a change that is closely associated with skin aging [12]. In addition to collagen, glycosaminoglycans (GAGs) and proteoglycans (PGs) are rich structural components of the extracellular matrix. These are all biological substances that are closely related to skin aging [13]. Therefore, it is extremely important to study the impact of collagen on skin aging, and each type contributes uniquely to the skin’s structural integrity and resilience. Among them, type I collagen, particularly the type 1A1 variant, is the most abundant, ensuring the skin’s strength and elasticity. However, with advancing age, the synthesis of type I collagen diminishes, leading to a reduction in the skin’s firmness and the emergence of wrinkles [14]. Furthermore, type III collagen, often associated with type I, plays a critical role during the early phases of wound healing, underscoring its importance in maintaining the skin’s repair mechanisms [15]. Another noteworthy collagen type, type IV, is found in the basement membrane zone, supporting the skin’s filtration and scaffolding functions [16]. The intricate balance between the synthesis and degradation of these collagen types is vital for skin health and its youthful appearance. Recent advancements in biotechnology have illuminated the significance of targeting specific collagen types in anti-aging treatments. For instance, interventions aiming at enhancing type I collagen synthesis can significantly ameliorate the visible signs of aging by improving the skin’s structural integrity. Similarly, protecting type IV collagen in the basement membrane can enhance the skin’s barrier function and resilience against environmental aggressors [17]. Hence, understanding the diverse roles of different collagen types in the skin not only enriches our knowledge of skin aging mechanisms but also opens new avenues for developing targeted anti-aging strategies. This nuanced approach allows for a more tailored intervention, potentially leading to more effective anti-aging treatments that can restore the skin’s youthful vigor by addressing the specific needs of its collagenous framework.

The following are the contents of collagen products and their impact on the resistance to skin aging.

When collagen is taken orally, it is digested and broken down in the gastrointestinal tract into smaller molecules, such as amino acids and small peptides, which are then absorbed through the gut and into the bloodstream. In the blood, these amino acids and small peptides are transported throughout the body, including the skin. Skin cells can use these amino acids to synthesize new proteins, including collagen [18,19] because amino acids are the basic building blocks of collagen and other proteins. In this way, oral collagen helps to supplement the loss of skin collagen, thereby improving the structure and appearance of the skin [18]. In a retrospective clinical trial on the effects of oral collagen, a total of 120 participants participated in a 90-day study. The subjects took collagen orally at prescribed doses every day. Participants’ skin was assessed by a variety of biomarkers and skin imaging techniques. The results showed significant improvement in the collagen fiber tissue of the vast majority of the subjects. In addition, many subjects also reported increased smoothness and elasticity of the skin and reduced wrinkles and fine lines [20]. In another study, which measured the moisture content of the stratum corneum and the rate at which water evaporated, the researchers found that oral collagen ingestion significantly improved the skin’s ability to retain moisture and reduced water loss. In addition, participants taking collagen also showed lower skin redness and inflammation during the study period compared to the control group. These findings further confirm that collagen can not only promote an improvement in skin structure but also enhance skin barrier function to resist the adverse effects of the external environment on the skin [21].

Ultraviolet radiation (UVR) plays a key role in skin aging, especially photoaging. UVR damages the skin through direct and indirect mechanisms, including DNA damage, promoting inflammatory responses, increasing the production of reactive oxygen species (ROS), and inhibiting the skin’s antioxidant defense mechanisms [22]. These changes lead to the degradation of collagen and elastin, which, in turn, affects the structure and elasticity of the skin and accelerates the appearance of skin aging. UVR also activates matrix metalloproteinases (MMPs) in the skin, which are responsible for degrading collagen and elastin, further damaging skin support structures [23]. In addition, the effects of UVR on skin and systemic homeostasis also include altering immune function, which may lead to the development of skin cancer. Therefore, protecting the skin from UVR damage is an important strategy to prevent and reverse skin aging [24]. Oral administration of antioxidant collagens (ACPs) has positive effects on photoaged skin structure and collagen. Serum-containing collagen peptide (CPS) and collagen peptide isolated from serum metabolite (SCP) can promote procollagen synthesis by activating the TGF-β/Smad pathway. Moreover, the expressions of AP-1, MMP-1, and MMP-3 proteins are inhibited to prevent collagen degradation; SCP can clear ROS and make the body more antioxidative; Ile-Hyp (IO) and Ala-Hyp-Gly (AOG) have been identified as active peptides that promote procollagen synthesis by activating the TGF-β/Smad3 pathway. Both are bioactive products that can be used as anti-skin-aging products [25].

Topical applications of collagen have been studied, although not as extensively as oral collagen. Previous studies have shown that topical collagen can improve skin elasticity and texture [26]. Topical collagen peptides directly affect fibroblasts, the cells in the skin responsible for collagen production. By stimulating these cells, local collagen can potentially enhance the skin’s natural collagen synthesis, improving firmness and reducing wrinkles [27]. However, it is important to note that because of its high molecular weight, collagen does not fully penetrate the skin when applied topically. This limitation may affect the efficacy of topical collagen therapy compared to other forms or applied approaches [28].

Related collagen products on skin aging were summarized in Table 1.

### 2.2. Enzymes

In addition to collagen, enzymes are also important for the skin. In recent years, protease has been found to be closely related to skin aging, and the current protease products are as follows.

Trypsin is a potent proteolytic enzyme that exhibits strong elastase activity by hydrolyzing peptide bonds, especially by breaking down specific amino acid sequences that are abundant in elastin. Elastin is the main component of elastic fibers, which provide stretch, recoil, and elasticity to the skin. Normal levels of elastic fiber production, organization, and integration with other cutaneous extracellular matrix proteins, proteoglycans, and glycosaminoglycans are integral to maintaining healthy skin structure, function, and youthful appearance [29]. In addition, elastin is a crucial protein within the skin’s extracellular matrix, and it is instrumental in bestowing the skin with its inherent elasticity and resilience. Elastin allows the skin to stretch and contract, a property fundamental to the skin’s dynamic nature [30]. The protein’s unique elastic properties stem from its ability to form extensive crosslinked networks, which confer the skin with the capacity to return to its original state postdeformation [3]. This feature is paramount not only for mechanical support but also for ensuring the skin’s durability over time. The aging process significantly impacts the synthesis and organization of elastin, leading to diminished skin elasticity and the onset of visible signs of aging, such as sagging and wrinkling. Ultraviolet radiation from sun exposure further exacerbates elastin degradation, compounding the effects of aging by promoting abnormal elastin accumulation and the formation of solar elastosis, a hallmark of photoaged skin.

To slow down skin aging, trypsin operates by effectively cutting and removing damaged or overcrosslinked elastin which is a significant feature in skin aging. This process helps to promote the synthesis and accumulation of new elastin, as removing old, damaged proteins can provide space and conditions for the production of new proteins. In addition, trypsin can improve the skin’s microcirculation and its ability to self-repair by cleaning up damaged proteins, thus countering the natural aging process [31].

Bromelain is a group of enzymes extracted from pineapple stems and fruits, which mainly includes a variety of proteolytic enzymes. It has a variety of pharmacological effects, including anti-inflammatory, antithrombotic, and fibrinolytic activities [32]. Bromoprotease reduces skin aging by operating according to a similar process as trypsin, albeit milder and more selective. It regulates the epidermal growth factor (EGF) signaling pathway, hydrolyzes dead skin cells on the skin’s surface, and removes proteins from the stratum corneum, thus promoting the regeneration of healthier skin cells [33]. The mechanism of action for elastin involves the selective hydrolysis of damaged tissues, which can selectively identify and decompose damaged or aged elastin, speeding up the skin repair process. To reduce skin stains, bromelain decreases melanin synthesis by affecting tyrosinase activity, which is mainly involved in regulating the microphthalmia-associated transcription factor (MITF) signaling pathway. MITF plays a crucial role in regulating tyrosinase expression in pigmentation cells [34].

Papain is an enzyme derived from papaya that reduces inflammation and removes dead skin cells that clog pores [35]. It is also able to remove damaged keratin that accumulates on the skin and forms small particles. Papain increases transepidermal water loss, degrades tight junction proteins, and causes vascular dilation [27]. When used locally, it exhibits high epidermal inflammatory potential by recruiting neutrophils, mast cells, and CD3-positive cells, and it induces Th2-biased antibody responses [36]. As we age, the hyaluronic acid polymers in the skin become more associated with the tissue. The proportion of hyaluronic acid released after papain digestion increases from 7% in the fetus to 23% in aging skin, but direct application of raw papaya or papain to the skin may cause irritation and even skin blisters [37]. 

Related enzyme products on skin aging were summarized in Table 2.

## 3. Polypeptides

Bioactive peptides are small protein fragments of 2 to 20 amino acids in length that occur naturally in food and have significant biological activity. These peptides have important pharmacological effects on the human body, such as anti-inflammatory, antibacterial, and antioxidant. Moreover, they can effectively inhibit the enzyme activity associated with chronic diseases and have shown the ability to significantly reduce the level of inflammatory factors in animal models [8]. These properties make bioactive peptides critical ingredients for maintaining skin health and reducing aging [38]. As natural products with specific biological activities, natural peptides play a crucial role in signal transduction and regulation of physiological processes. They are regarded as biological compounds with potential, which can not only alleviate the early aging phenomenon of skin and strengthen the skin barrier function but also provide protection against ultraviolet radiation [39]. Bioactive peptides also have the effect of promoting skin regeneration. Existing studies have shown that new materials rich in bioactive peptides exhibit good cell compatibility with keratinocytes and fibroblasts and can promote skin regeneration and further improve skin function [40,41]. At present, polypeptides mainly exert their effects in the following aspects.

### 3.1. Promote Collagen Synthesis

One of the primary roles of bioactive peptides in skin regeneration involves the stimulation of collagen synthesis. Peptides such as copper tripeptide-1 and palmitoyl pentapeptide-4 are known to upregulate collagen production in fibroblasts, the principal cells responsible for extracellular matrix synthesis [42]. This not only aids in reducing the appearance of wrinkles but also enhances skin firmness and integrity, counteracting the effects of aging. Bioactive peptides can modulate the activity of enzymes involved in skin matrix remodeling, such as matrix metalloproteinases (MMPs). By inhibiting MMP activity, these peptides prevent the degradation of collagen and elastin, thereby preserving the skin’s structural framework and elasticity [43]. Additionally, some peptides exhibit antioxidative properties, protecting skin cells from oxidative stress-induced damage by neutralizing reactive oxygen species (ROS) [44,45]. This antioxidative defense mechanism is vital for maintaining cellular health and preventing premature aging. The role of bioactive peptides extends to enhancing epidermal barrier function and hydration. Peptides like acetyl hexapeptide-8, known for its moisture-binding properties, can improve skin hydration by enhancing the expression of hyaluronic acid, a natural humectant [46]. This not only helps in maintaining skin’s moisture balance but also supports the barrier function, protecting against environmental stressors.

Specific peptides, such as the aminoacyl tRNA synthase complex-interacting multifunctional protein 1 (AIMP1)-derived polypeptide (AdP, INCI named Sh-oligopeptide-5/sh-oligopeptide SP) have been shown in experiments to have a positive effect on type I collagen synthesis in human fibroblasts, indicating its anti-skin-aging efficacy [47].

GEKG, a tetrapeptide extracted from the extracellular matrix (ECM), has been shown in studies to have a significant effect on human fibroblasts, significantly promoting collagen production and reducing skin roughness [48].

### 3.2. Inhibit Collagen Degradation

Chickpea seed peptide (CSP) has the ability to inhibit elastase and tyrosinase, which are involved in the skin aging process. By regulating the activity of these enzymes, CSP is able to slow down the degradation of skin collagen, thus playing a key role in preventing collagen loss and maintaining skin elasticity and firmness [49].

Salvia hispanica peptides are well known for their excellent antioxidant ability to lower blood pressure and anti-inflammatory activities. More specifically, chia seed peptides smaller than 3000 daltons have been shown to significantly inhibit key skin enzymes, such as collagenase, hyaluronidase, tyrosinase, and elastase, thus ultimately helping to prevent skin aging [50].

Collagen peptides extracted from fish scales are called fish scale collagen peptides (CPNS), and they are composed of a Gly-Pro sequence, which is the key active ingredient in CPNS. This peptide can effectively inhibit the production of matrix metalloproteinase MMP-1, which helps to enhance the structural stability and elasticity of the skin, thus combating skin aging [51].

Five low-molecular-weight peptides (GGFDLs) in low-molecular-weight (LMW) feather keratin hydrolysate inhibit the expression of MMP-1 acting as novel bioactive compounds that protect the skin from internal and external factors [52].

### 3.3. Boost Antioxidant Defenses

One cosmetic product utilizes a medicinal peptide (arnosine + acetyl tetrapeptide-5 + hexapeptide-11 + acetyl hexapeptide-3 (HEXAPepTIde-3)). This peptide is effective in significantly reducing the content of malondialdehyde and oxygen free radicals in skin cells. Additionally, it increases the content of hydroxyproline and collagen, improves the activity of the superoxide dismutase (SOD) enzyme and glutathione (GSH) enzyme, and demonstrate a positive anti-aging effect on the skin [53].

### 3.4. Enhance Autophagy

Neprilysin (NEP) is an endogenous peptide composed of ten amino acids (sequence N-YGGFLRKYPK-C) that exhibits significant anti-aging effects in skin cells. The main anti-aging mechanism of NEP is through activation of autophagy which helps to reduce ultraviolet B (UVB)-induced skin aging. Autophagy is an intracellular process that removes damaged cellular components and is essential for maintaining the health and function of skin cells [54].

### 3.5. Inhibit Melanin Formation

Crocodile white blood cell extract (cWBC) is a rich source of bioactive peptides. These peptides have remarkable anti-aging properties, especially in inhibiting melanocyte deposition induced by UVB radiation. Melanosis is an important sign of skin aging, so cWBC extract helps to slow down the aging process of the skin by inhibiting this process [55]. Melanin, a natural pigment found in skin, hair, and eyes, is responsible for giving skin and hair their color and plays a key role in protecting skin from ultraviolet (UV) damage [56]. The process of melanin formation is called melanopoiesis and is mainly carried out in the melanocytes of the skin. When the skin is exposed to UV radiation, it stimulates melanocytes to produce more melanin, which acts as a natural protective mechanism to absorb and disperse UV rays, thereby protecting the skin from damage. However, with age and excessive exposure to UV light, excessive deposition of melanin can lead to uneven pigmentation, such as spots and increased pigmentation, which are common signs of skin aging [57]. Therefore, by inhibiting the activity of melanocytes induced by UVB radiation, the application of cWBC extract can not only help slow down the skin aging process but also improve the overall color and health of the skin.

### 3.6. Enhanced Barrier and Repair Effects

T14 is a 14-amino-acid peptide derived from the C-terminal of acetylcholinesterase (AChE). It has been found to effectively promote skin renewal and metabolism, which plays an important role in maintaining the skin’s youthful state. T14 reduces aging through its main defense process by strengthening the skin barrier, improving skin elasticity, and promoting the repair of damaged tissues. These effects help maintain the structure and function of the skin, thereby resisting the skin aging process [58].

### 3.7. Promotion of Collagen Synthesis, Inhibition of Collagen Degradation, and Anti-Inflammatory, Antioxidant, and Other Comprehensive Effects

GHK-Cu (glycyl-L-histidyl-L-lysine-Cu) is a peptide that occurs naturally in human blood serum and is widely used in the field of skin care, where in vitro and in vivo studies have shown that GHK-Cu promotes skin remodeling, wound healing, and regeneration, and has significant antioxidant and anti-inflammatory effects [59]. Cyanocuprin promotes collagen and elastin synthesis by increasing fibroblast activity, regulated by transcription factors such as Smad and mitogen-activated protein kinase (MAPK) pathways [60]. Increased collagen and elastin help improve the structural strength and elasticity of the skin and reduce the formation of wrinkles. Blue copper peptides are involved in skin extracellular matrix remodeling by regulating the expression of matrix metalloproteinases (MMPs) and tissue inhibitors (TIMPs). In addition, it is characterized by significant anti-inflammatory effects which mainly inhibit pro-inflammatory cytokines produced by inflammatory cells such as macrophages. GHK-Cu treatment primarily inhibits NF-κB p65 and p38 in the MAPK signaling pathway, which reduces the production of ROS, increases the activity of SOD, and decreases the production of tumor necrosis factor alpha (TNF-α) and interleukin 6 (IL-6). These combined actions can reduce skin damage and aging caused by inflammation [61]. Polypeptide ingredients have been highly valued by skin care brands for their remarkable effects in skin care. More specifically, the application of peptides in high-end skin care brands has become a popular trend. GHK-Cu is found in certain brands of anti-aging creams and serums, which have been developed based on in-depth skin science research to reduce wrinkles and improve skin quality by promoting collagen synthesis, inhibiting its degradation, and enhancing the skin’s repair and defense capabilities.

The NF-κB pathway is directly related to skin aging caused by UVB-induced oxidative stress damage, and NF-κB mobilizes UVB causing skin inflammation due to oxidative stress [62].

Related peptide products on skin aging were summarized in Table 3.

## 4. Amino Acids

Amino acids are a key ingredient in skin care and anti-aging products, and they are widely used because of their properties as natural moisturizing factors. Amino acids, such as arginine and glycine, not only play a pivotal role in skin hydration, but are also regarded as building blocks for the synthesis of skin proteins, such as collagen and elastin [63].

Arginine improves blood flow and accelerates wound healing and collagen production, which, in turn, help maintain and repair the skin barrier [63]. Arginine shows antioxidant activity that helps neutralize free radicals. Free radicals are unstable molecules that cause premature skin wrinkles and fine lines. As a moisturizer, arginine regulates skin hydration and is involved in the synthesis of components of the skin’s natural moisturizing factors (NMFs) such as ceramides, cholesterol, urea, and glycosaminoglycans. One study evaluated the effect of 2.5% arginine hydrochloride ointment on water loss from the top layer of the skin and found that topical application of arginine increased urea content and improved skin hydration [64].

Glycine is the most abundant amino acid in collagen and is essential for maintaining the skin’s elasticity and structural integrity [64]. Glycine is believed to help support the production and stimulation of healthy collagen and to be able to penetrate the skin as one of the smallest molecular amino acids. In skin care, glycine is mainly used to improve moisturizing ability, increase collagen production, and promote skin repair and regeneration. These properties make it a valuable ingredient in anti-aging skin care products [65]. In genetically diverse mice, glycine was found to boost lifespan, suggesting it may play a role in extending it. This effect has also been observed in other model organisms, further demonstrating that glycine may help improve overall health during aging [66]. Supplementation with a combination of glycine and n-acetylcysteine (GlyNAC) has been shown to have positive effects on aging in older adults, including improving GSH deficiency, alleviating oxidative stress, improving mitochondrial function, lowering inflammation, improving body function, and slowing markers of aging [67]. 

Related amino acid products on skin aging were summarized in Table 4.

## 5. Cell-Derived Products

Skin cells play an important role in the field of skin aging research. Many clinical experiments and screenings for anti-skin-aging drugs rely on these skin cells. At the same time, skin cells exhibit biological activity, producing bioactive factors. These active cells and factors also play a role in skin aging treatment. In addition, the interaction between cells and the skin microenvironment is crucial in this process. The current cell-derived products include stem cells and exosomes.

Adipose-derived stem cells (ASCs) are important stem cells isolated from adipose tissue. ASC-derived exosomes (ASC-exos) are important components released by paracrine action and exhibit various biological activities [69]. ASC-exos have the ability to promote tissue regeneration and can be used in the anti-aging treatment of skin [70]. Adipose-derived stem cells (ADSCs) can secrete a series of growth factors and cytokines, such as hepatocyte growth factor (HGF) and vascular endothelial growth factor (VEGF), and promote growth inflammatory cytokine IL-6. Direct evidence of ADSCs’ paracrine effect was observed in studies investigating the promotion of human dermal fibroblast (HDF) function by adipose-derived stem cells (ADSCs). Through this mechanism, ADSCs can promote the proliferation of HDF, increase the expression of type I collagen, and decrease the expression of MMP-1. However, the same study also showed that the paracrine effect of ADSCs increased the mRNA expression of SA-β-Gal associated with aging, suggesting the acceleration of the aging process [71]. The influence of ADSCs on the skin microenvironment is mainly reflected in the regulation of the behavior of surrounding cells through the secretion of growth factors and cytokines. For example, they promote angiogenesis and improve the blood supply to the skin by releasing HGF and VEGF, thus enhancing the nutrient and oxygen supply to the skin cells. Although the pro-inflammatory cytokine IL-6 secreted by ADSCs can promote cell regeneration in the short term, it may lead to the acceleration of chronic inflammation and aging in the long term [72].

Extracellular vesicles (EVs) released by gingival-derived mesenchymal stem cells (GMSC-EVs) inhibited cellular senescence induced by oxidative stress in human endothelial cells and skin fibroblasts. These Evs can reduce the positive rate of β-galactosidase and the expression of aging proteins p21 and p53, and they can significantly improve skin aging caused by oxidative stress [73]. As a key carrier of intercellular communication, extracellular vesicles affect the skin aging process by regulating the age-related secretory phenotype (SASP) of cells [74].

Exosomes are paracrine products of mesenchymal stem cells that have shown potential to regulate immune response and promote cell proliferation, wound healing, and migration, as well as anti-aging [75]. Exosomes can influence cellular pathways associated with aging, such as by activating the AKT and ERK signaling pathways, which are associated with cell survival, proliferation, and anti-aging. Activation of AKT and ERK can promote cell survival and division and inhibit programmed cell death. Specific miRNAs and proteins in exosomes can influence cell cycle regulators, such as p53, p21, and p16INK4a, potentially reversing cell aging [76]. Chronic inflammation is another key driver of aging. Anti-inflammatory molecules in exosomes can regulate inflammatory response and reduce chronic inflammatory states by reducing the release of pro-inflammatory cytokines [77].

Chronic inflammation is another key driver of aging. Anti-inflammatory molecules in exosomes can regulate inflammatory response and reduce chronic inflammatory states by reducing the release of pro-inflammatory cytokines [75]. These anti-inflammatory molecules regulate the activity of immune cells by affecting cell signaling and gene expression. They can inhibit the production of pro-inflammatory factors, such as TNF-α and IL-6, while promoting the release of anti-inflammatory factors, such as interleukin 10 (IL-10). This regulatory effect helps to restore the balance among cells and reduce the damage of inflammatory responses to tissues [78].

The potential usage of ascorbic acid (AA)-supplemented stem cell secretomes (SCS). The effectiveness of SCS in improving skin conditions has been confirmed in in vivo and in vitro studies, indicating the essence of SCS application in alleviating the process of skin aging [79]. EVs from plants, as a plant-derived bioactive factor, have shown their ability to act as skin antioxidants, providing another strategy to improve and prevent skin-aging-related phenomena [80]. Through direct interaction with skin cells, they stimulate collagen and elastin syntheses while inhibiting oxidative stress caused by UV light or other environmental factors. In addition, SCS can also improve the skin’s microcirculation and enhance its ability to repair itself, which is especially important for preventing early skin aging.

EVs composed of lipid bilayers can be obtained from a variety of pathways, both in plant cells and microorganisms, and are important mediators of intercellular communication. Plant-derived EVs are bioactive factors derived from plants, which can be used as skin antioxidants. They help to resist skin oxidation and improve skin aging [81]. Plant-derived EVs contain a variety of antioxidants, such as polyphenols and vitamins, which neutralize free radicals and reduce oxidative stress, thereby protecting skin cells from damage caused by environmental stress. In particular, vitamin D is an important fat-soluble vitamin that has multiple benefits for skin health. It not only supports the immune function of the skin but also helps maintain the integrity of the skin barrier and promotes cell growth, repair, and metabolism [82]. Vitamin D regulates more than 200 genes by binding to the vitamin D receptor (VDR) in skin cells, including those involved in cell proliferation, differentiation, and immune and barrier functions [83]. Therefore, the vitamin D contained in EVs can slow down the skin aging process by acting directly on skin cells to enhance the skin’s resistance to environmental factors. In addition, these EVs can also enhance skin barrier function by regulating skin cell signaling pathways and promoting cell proliferation and differentiation [81]. EVs can also provide a way to mimic nature to enhance skin defense mechanisms by mimicking cellular communication mechanisms in natural ecosystems, which is particularly important for protecting skin under increasingly harsh environmental conditions [84]. 

Related cell products on skin aging were summarized in Table 5.

## 6. Products of Microbial (Bacterial and Fungal) Origin

In recent years, skin microbes have demonstrated a significant impact on the process of skin aging, leading to the rapid development of related products. The main research and developments are presented below.

### 6.1. Germ

The microbiome balance of the skin plays a vital role in its health and aging state. Recent research has revealed that specific bacterial communities on the skin can promote the health of the skin and are negatively correlated with its rate of aging. For example, certain probiotics, when applied to the skin, fight free radicals by producing natural antioxidant enzymes, anti-inflammatory molecules, or other bioactive substances that are beneficial to the skin.

The study found that *Lactobacillus plantarum* showed good results in skin care. The treated skin significantly improved in moisture, elasticity, and overall skin appearance [85]. Marine microorganisms are also an important source of probiotic research. Wound closure of the skin is a medical problem related to skin aging, which is achieved by transforming lactobacilli into plasmids encoding CXCL12. CXCL12 can increase the proliferation of dermal cells and macrophages to enhance wound closure when administered locally to wounds of mice, and accelerating wound healing can improve bioavailability and prevent skin aging [86].

The bioactive extract of *Sphingomonas hydrophobicum* (SH) (SA223-S2BM) has the effect of preventing skin aging. SA223-S2BM reduces oxidative stress by activating intracellular protein inhibition modules and helps maintain the stability of the proteome. It can significantly inhibit the activity of β-galactosidase and the expression of the p21 and p16 proteins, which is essential in inhibiting skin aging [87].

The bacterium chlorophyll is isolated from photosynthetic bacteria *Rhodobacter sphaeroides* and has been demonstrated to have anti-aging effects on the skin. *Bacterium chlorophyll* operates through the enhancement of procollagen production, antioxidant, and anti-inflammatory activities. Moreover, bacterial chlorophyllin has not only demonstrated excellent gene expression in promoting skin cell differentiation and growth, but it has also shown potential in improving the expression of genes related to skin barrier function [88].

The T65 strain demonstrated inhibition against a wide range of human pathogenic bacteria and was excellent at eliminating 1,1-diphenyl-2-picrylhydrazyl (DPPH) free radicals and elastase activity. This makes it a potential natural substance in the anti-skin-aging, anti-oxidation, and whitening fields [85].

Marine bacteria also offer a new strategy for skin care. The secondary metabolites produced by them have strong antioxidant and UV protection capabilities. For example, certain bacteria from the deep ocean can produce specific peptides that have a natural UV protection effect on the skin, preventing the damage caused by daily UV rays and, thus, delaying skin aging.

The application of live bacteria, particularly from the *Lactobacillus* and *Bifidobacterium* genera, in skin care has garnered attention for their ability to enhance skin barrier function, modulate the immune response, and inhibit the growth of pathogenic bacteria. For example, topical formulations containing *Lactobacillus plantarum* have demonstrated efficacy in reducing skin inflammation, accelerating wound healing, and improving skin hydration and elasticity [89]. These probiotic treatments can be particularly beneficial for conditions like atopic dermatitis, acne, and rosacea, offering a natural and gentle approach to managing skin health.

### 6.2. Fungus

*Aspergillus pheasicus* is a probiotic fungus that occurs naturally in traditional Chinese fermented teas such as Fuzia tea and Pu-erh tea, also known as golden flower fungus (GFF). Studies have found that ginseng extract (GFFG) fermented by GFF has significant anti-aging effects on skin cells. GFFG can promote the synthesis of hyaluronic acid in skin cells, which is the skin’s natural moisturizing factor and is essential for maintaining skin elasticity and moisture [90]. Hyaluronic acid (HA) is one of the main components of the extracellular matrix, which is widely distributed in the human body. Because of its unique physical and chemical properties and diversity of physiological functions, hyaluronic acid is widely used in tissue engineering and regenerative medicine [91]. In addition, GFFG inhibits TNF-α-induced expression of MMP-1 in skin fibroblasts, an enzyme that breaks down collagen and is a key protein in maintaining skin structure and elasticity. Therefore, through this process, GFFG helps to slow the aging of the skin and prevents the development of wrinkles [92].

In addition to *Aspergillus cockscomb*, other fungal species have also shown potential for promoting skin health. Metabolites of certain yeasts (such as *Saccharomyces*) and molds (such as Penicillium) contain peptides and polysaccharides, which act as natural antioxidants to protect the skin from free radical damage while promoting collagen synthesis to combat wrinkle formation. The antioxidant components of yeast extracts, particularly *Saccharomyces cerevisiae*, activate nuclear factor erythroid-derived 2-like 2 (Nrf2), a transcription factor that plays a key role in the oxidative stress response. Activation of Nrf2 helps to upregulate the expression of antioxidant enzymes, such as SOD and glutathione peroxidase (GPx), protecting cells from free radical damage [86].

Microbial-fermented products, such as tea fermented by *Aspergillus cristatus*, have shown their health benefits in Fuzhuan tea and pu-erh tea in China. This type of fungus is called GFF, and its unique fermentation effect not only enhances the taste of tea but is also rich in microbial metabolites that are beneficial to the human body. Studies have shown that GFF solid fermented ginseng extract (GFFG) has several advantages including promoting anti-aging effects, enhancing the skin’s moisturizing function, promoting the synthesis of hyaluronic acid, enhancing the expression of aquaporin, and increasing the level of filagrat mRNA in skin cells [90]. These activities effectively combat the aging process of the skin by reducing the expression of inflammatory factors, such as TNF-α-induced skin MMP-1 [92].

Fermented skin care products, utilizing bacterial and fungal metabolites, are rich in enzymes, vitamins, and antioxidants that promote skin health. For instance, the fermentation of sea kelp by *Bacillus* sp., yields a high concentration of fucoidan, a compound known for its anti-inflammatory and antioxidant properties [93]. Such fermentation products can enhance skin hydration, elasticity, and overall complexion, contributing to their anti-aging effects.

Furthermore, existing research has also shown that microorganisms have many widespread applications in the field of skin aging and regeneration. For example, astaxanthin is a pigment derived from microorganisms that has the ability to scavenge oxygen free radicals and has always had an effect on skin aging. Solid lipid nanoparticles containing astaxanthin, nanostructured lipid carriers, and polymer nanospheres are often considered new materials that have good anti-skin-aging effects, and this application has broad application prospects [94].

Related bacterial and fungal products on skin aging were summarized in Table 6.

## 7. Nucleic Acid

Studies have shown that nucleic acids and their derivatives can be used as active ingredients in skin care products to help repair damaged DNA and protect skin from UV and free radical damage [96]. This protective effect is primarily accomplished by enhancing the self-repair capability of cells. The process involves increasing the activity of nucleic acid repair enzymes, which are crucial for averting the loss of cell function and mitigating skin aging caused by DNA damage [97].

RNA molecules, especially small RNAs (such as microRNAs), also play an important role in regulating the process of cell aging. These small molecules can affect the life cycle of skin cells by regulating gene expression and, thus, affect the aging of skin [98]. For example, specific microRNAs are able to regulate collagen and elastin synthesis, which are key components of skin elasticity and firmness [99].

Some molecules derived from nucleic acids, such as nucleotides and nucleosides, have been shown to have anti-aging effects. They can be used as molecules of cellular energy and signal transmission to improve the metabolic state of cells [100]. This is especially important for maintaining the vitality of skin cells and resisting the stress of aging.

Exogenous nucleotides and nucleosides can act on purine receptors of skin cells to activate a series of signaling pathways, which are related to skin cell proliferation, differentiation, and immune regulation. This signal transduction mechanism plays an important role in the skin’s immune and inflammatory responses and may have a positive impact on preventing the process of premature skin aging [101].

Related nucleic acid products on skin aging were summarized in Table 7.

## 8. Discussion and Summary

Bioactive ingredients play an important role in delaying and fighting skin aging. From the perspective of molecular biology, skin aging is a multifactor, multistep complex process involving gene expression, protein synthesis, cell metabolism, and others. In this process, collagen, peptides, enzymes, amino acids, and cell secretory products, including microbial interactions and other bioactive molecules and related products play a central role.

As a key protein in skin elasticity and structure, the balance between the synthesis and degradation of collagen has a direct impact on skin aging. At present, collagen itself, including many peptides, plays a very important role in this respect. Product lines are rich, featuring important anti-aging strategies that work to elevate collagen levels, fortify antioxidant defenses, and enhance the self-repair ability of the skin.

At the cellular level, bioactive factors, such as stem cell factors, exosomes, and small RNAs, have the potential to regulate the behavior of skin cells, and combat skin aging by influencing the cells’ antioxidant defense system and promoting collagen synthesis. These molecules regulate signaling pathways, promote cell cycle progression, and enhance DNA repair capabilities, thereby inhibiting age-related decline in cell function.

At present, research on the role of microorganisms in skin aging is also developing very rapidly, and studies on probiotics such as *Lactobacillus* have shown that they can improve skin barrier function, improve the skin’s moisturizing ability, and may have a positive effect on immune regulation. In addition, the application of antioxidant chlorophyll produced by Marine bacteria in UV protection demonstrates the great potential of microbial products in anti-aging protection.

Recent research findings show that bioactive ingredients are increasingly becoming important in skin anti-aging and interest in this field continue to grow rapidly. These new resource products may provide us with new, effective anti-aging ingredients. This will not only promote basic scientific research but also bring innovative ingredients to the skin care industry to meet the market demand for healthy and youthful skin. Future research work can be focused on the following three aspects: First, future studies can focus on the effects of enhancing collagen-related peptides on skin aging. Second, another interesting line of research would be to explore cell-derived factors, such as stem-cell- and exosome-related factors and small RNA regulation in skin aging. Lastly, it is important to develop more bacterial strains and other microbial resources from the perspective of microorganisms and explore the effects of microbial metabolites on skin aging to find new anti-aging bioactive molecules.

**Figure 1:**
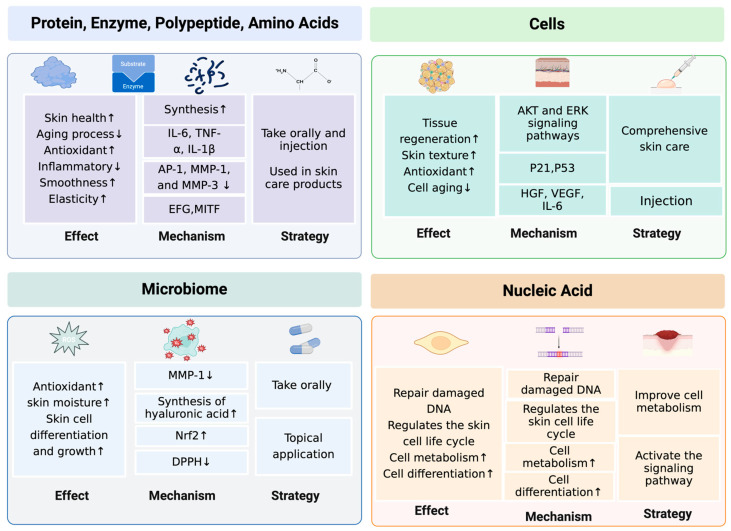
Mechanisms of action of anti-aging bioactive factors and related strategies. ↑, increase; ↓, decrease.

Building on previous research, our current study not only delved into the mechanisms of skin aging bioactive factors but also identified and characterized novel bioactive compounds and their therapeutic potential (Figure 1). Given that our previous work provided a comprehensive review of metabolic pathways and their effects on skin aging, this study enriches this discourse by highlighting recent advances in bioactive factors, highlighting innovations in extraction techniques, formulation strategies, and their efficacy in combating skin aging. Specifically, we present novel bioactive factors from emerging natural sources, explore their synergies in multicomponent formulations, and provide data from recent clinical trials confirming their benefits. This advance represents a major leap forward in alleviating skin aging through bioactive compounds and demonstrates our commitment to evolving research that addresses the complex mechanisms of skin aging. 

## Figures and Tables

**Table 1:** Effects and mechanisms of collagen and related substances on skin aging.

Source	Substance	Dosage	Method of Administration	Experimental Material	Role	Mechanism	References
Animals (cows, chickens, pigfish)	Collagen	2.5 g/d to 10 g/d (collagen hydrolysate), 3 g/d (collagen tripeptide)	Oral	Human skin	Improves skin quality, improves smoothness and elasticity, and wrinkles and fine lines are reduced	Collagen fibrous tissue ↑, skin moisturizing ability ↑, water loss ↓, skin redness and inflammation ↓	[20,21]
Animals (cows, chickens, pigfish)	Collagen hydrolysate		Oral	Uva-induced human skin fibroblasts (ESFs)	Skin structure is improved and collagen synthesis is promoted	Activating the TGF-β/Smad pathway, AP-1, MMP-1, and MMP-3 express ↓, free radical content ↓	[25]

↑, increase; ↓, decrease.

**Table 2:** Effects and mechanisms of enzymes on skin aging.

Source	Substance	Dosage	Method of Administration	Experimental Material	Role	Mechanism	References
Biological enzyme	trypsin	1 micromole	External use	Human abdominal skin biopsy samples from different age groups (young, adult, elderly)	Skin microcirculation and self-repair ability ↑	Cut and remove damaged or overcrosslinked elastin; synthesis and accumulation of new elastin ↑	[31]
Biological enzyme	bromelain		Oral	Animal	Anti-inflammatory; melanin synthesis ↓	Regulation of the epidermal growth factor (EGF) signaling pathway; regulation of the MITF signaling pathway; identification and break down of damaged or old elastin	[32,33,34]
Biological enzyme	papain	0.1 to 2.0 mg/mL	External use	Anti-inflammatory; dead skin cells that clog pores; keratinocytes, casein, collagen, keratin, and pig skin	Anti-inflammatory; dead skin cells that clog pores	Hydrolysis of skin surface proteins ↑, degradation tight junction protein ↑, skin cell renewal ↑	[27,35,36,37]

↑, increase; ↓, decrease.

**Table 3:** Effects and mechanisms of peptides related to skin aging.

Source	Substance	Dosage	Method of Administration	Experimental Material	Role	Mechanism	References
Cosmetics	AIMP1-derived peptide (AdP)		Oral, external use	Elderly mouse models and mouse models with epidermal-specific Pgc-1α gene knockout (epiPgc-1α KO)	Skin elasticity and moisture ↑	Human fibroblast type I collagen synthesis ↑	[47]
Extracellular matrix	GEKG		In vitro study, external use	In vitro model: human dermal fibroblast; in vivo model: human body	Smooths skin texture; reduces rough skin; skin appearance and feel ↑	Collagen production ↑	[48]
Bird seed	Canary seed peptides (CSPs)	6.2 mg/mL, 6.1 mg/mL	Computer simulation	Characterization in silicon, molecular dynamics simulation	Regulation of collagen metabolism	Elastase and tyrosinase ↓; skin collagen degradation ↓;	[49]
Chia seed	Chia seed peptide	2.5 mL/kg/day and 5 mL/kg/day (animal); 50 g/day (human)	Oral	Human body, animal	Antioxidant, anti-inflammatory	collagenase ↓; hyaluronidase ↓; collagen and elastin breakdown ↓	[50]
Fish scale	CPNS	0.5, 1, 5, 10, and 20 μg/mL	In vitro study, oral	Human skin cells	Skin structure stability and elasticity ↑;	MMP-1 ↓; collagen synthesis ↑	[51]
Feather	LMW feather keratin hydrolysate	1000 mg/day	Oral	Human	protects the skin from both internal factors (natural aging processes, genetic factors, changes in hormone levels, and the production of free radicals in the body) and external factors (ultraviolet (UV) radiation, pollution, chemical exposure, and changes in temperature and humidity)	MMP-1 ↓; collagen synthesis ↑; antioxidant capacity ↑; inflammatory mediators ↓	[52]
Endogenous decapeptide	NEP	5 μg/mL	In vitro study	Human dermal fibroblast cell line Hs68	Skin appearance and feel ↑	Activation of autophagy through the mTOR-Beclin-1-mediated signaling pathway; collagen content ↑; MMPs activity ↓	[54]
Crocodile leukocyte	Crocodile white blood cell extract	6.25–400 μg/mL	In vitro study, external use	Ultraviolet B (UVB) irradiation of melanocytes	Melanin formation ↓	UVB-induced melanosis ↓	[55]
Endogenous peptide	T14		In vitro study	Human keratinocytes	Cell growth and renewal ↑	Allosteric sites that bind alpha-7 receptors influence calcium inflow	[58]
Human serum	GHK-Cu	10 mg/mL, 0.05% topical cream	Injection for topical use	Human	Skin remodeling and regeneration ↑; synthesis of collagen and elastin ↑; antioxidant capacity ↑	Smad and MAPK ↑; NF-κB p65 and p38 MAPK pathways ↓; TNF-α and IL-6 ↓; regulates the expression of MMPs and TIMPs	[59,60,61]

↑, increase; ↓, decrease.

**Table 4:** Effects and mechanisms of amino acids related to skin aging.

Source	Substance	Dosage	Method of Administration	Experimental Material	Role	Mechanism	References
Amino acid	Arginine	2.5% arginine hydrochloride ointment	External use	Human	Skin elasticity and barrier function ↑, maintains skin barrier	Collagen production ↑	[64,65]
Amino acid	GlyNAC		Oral	Human	Maintains skin elasticity and structural integrity	Collagen synthesis ↑, antioxidant capacity ↑	[67,68]

↑, increase; ↓, decrease.

**Table 5:** Influence of cells on skin aging and its mechanism.

Source	Substance	Method of Administration	Experimental Material	Role	Mechanism	References
Adipose tissue	ASCs	Injection, culture in vitro	Animal, in vitro studies	Tissue regeneration	Promote tissue regeneration through ASC-derived exosomes (ASC-exos); improve skin structure	[69,70]
Adipose tissue	ADSCs	Injection, topical application	Animals, cells, humans	Enhanced skin cell function	Secretion of growth factors and cytokines (HGF, VEGF, IL-6); dermal fibroblast function ↑; type I collagen expression ↑	[69,71]
Gum	GMSC-EV	Injection, culture in vitro	Animal, cells	Inhibition of cellular senescence induced by oxidative stress	β-Galactosidase positive rate ↓; expression of aging proteins p21 and p53 ↓	[73,74]
Mesenchymal stem cells	Mesenchymal stem cell exosomes	Injection, topical application	Animals, cells, humans	Immune response regulation, cell proliferation and migration promotion	Regulate the immune response; cell proliferation and migration ↑; anti-aging by activating the AKT and ERK signaling pathways; contain specific miRNAs and proteins that affect cell cycle regulators	[75,76,77]
Stem cells	SCS	Injection, topical application	Animal, cells	Skin condition improvement	The ability of cells to repair themselves ↑; antioxidant capacity ↑	[79]
Plant cells and microorganisms	EVs	Injection, topical application	Animals, cells, humans	Skin antioxidant	Antioxidant ability ↑	[80,81]

↑, increase; ↓, decrease.

**Table 6:** Effects and mechanisms of bacterial and fungi on skin aging.

Source	Substance	Dosage	Method of Administration	Experimental Material	Role	Mechanism	References
Probiotics	*Lactobacillus plantarum*	100 ng/mL	External use	Caco-2 cells	Skin moisturizing, elasticity, and looks ↑	Produces natural antioxidant enzymes, anti-inflammatory molecules, and other beneficial substances	[85]
Marine microorganism	*Sphingomonas hydrophobicum*		External use	Full-thickness skin equivalent model of aging	Prevent skin aging	Activates the protein inhibition module; oxidative stress ↓	[87]
Photosynthetic bacteria	*Rhodobacter sphaeroides*	0.001% (*w*/*w*)	External use	Human dermal fibroblasts and keratinocytes	Skin cell differentiation and growth ↑	Procollagen production ↑; antioxidant and anti-inflammatory activity ↑	[88]
Germ	T65	250 pg/mL	External use	CCD-986Sk cells Skin cells, dermal cells, and macrophages	Antioxidant capacity ↑, whitening	Human pathogenic bacteria ↓; DPPH free radical ↓; elastase activity ↓	[85]
Germ	The plasmid encoding CXCL12 transforms *Lactobacillus*		Oral	CCD-986Sk cells Skin cells, dermal cells, and macrophages	Skin cell differentiation and growth ↑	Proliferation of dermal cells and macrophages ↑	[95]
Fungus	Yeasts and molds		External use	skin	Skin sparing	Contains peptides and polysaccharides that act as natural antioxidants; activates Nrf2 transcription factors; collagen synthesis ↑	[86]
Fungus	GFF	100 MCG/mL	External use	Keratinocytes, human dermal fibroblasts	Skin moisturizing function ↑	Synthesis of hyaluronic acid ↑; expression of aquaporins ↑; MMP-1 expression induced by inflammatory factors such as TNF-α ↓	[90,92]

↑, increase; ↓, decrease.

**Table 7:** Effect and mechanism of nucleic acid related to skin aging.

Source	Substance	Dosage	Role	Mechanism	References
Exogenous and endogenous	Nucleic acids and their derivatives	100 nm	Repair damaged DNA, protect skin	Cell self-repair ability ↑, activity of nucleic acid repair enzymes ↑	[96,97]
Exogenous and endogenous	Small RNAs (e.g., microRNA)		Regulates the skin cell life cycle	Regulating gene expression, synthesis of collagen and elastin ↓	[98,99]
Exogenous and endogenous	Nucleotides and nucleosides	1 micromole to 50 micromole	Improves cell metabolism	As a cellular energy and signaling molecule, it maintains the vitality of skin cells	[100]
Exogenous	Exogenous nucleotides and nucleosides		Skin cell proliferation, differentiation and immune regulation	It acts on purine receptors and activates signaling pathways related to skin cell proliferation, differentiation, and immune regulation	[101]

↑, increase; ↓, decrease.

## Data Availability

Data are contained within the article.
